# PD-L1 upregulation in myeloma cells by panobinostat in combination with interferon-γ

**DOI:** 10.18632/oncotarget.26726

**Published:** 2019-03-08

**Authors:** Masami Iwasa, Takeshi Harada, Asuka Oda, Ariunzaya Bat-Erdene, Jumpei Teramachi, Hirofumi Tenshin, Mohannad Ashtar, Masahiro Oura, Kimiko Sogabe, Kengo Udaka, Shiro Fujii, Shingen Nakamura, Hirokazu Miki, Kumiko Kagawa, Shuji Ozaki, Masahiro Abe

**Affiliations:** ^1^ Department of Hematology, Endocrinology and Metabolism, Institute of Biomedical Sciences, Tokushima University Graduate School, Tokushima, Japan; ^2^ Department of Tissue Regeneration, Institute of Biomedical Sciences, Tokushima University Graduate School, Tokushima, Japan; ^3^ Department of Orthodontics and Dentofacial Orthopedics, Tokushima University Graduate School of Oral Sciences, Tokushima, Japan; ^4^ Division of Transfusion Medicine and Cell therapy, Tokushima University Hospital, Tokushima, Japan; ^5^ Department of Hematology, Tokushima Prefectural Central Hospital, Tokushima, Tokushima, Japan

**Keywords:** multiple myeloma, panobinostat, PD-L1, STAT1, IFN-γR1

## Abstract

Immunotherapy is revolutionizing the treatment paradigm for multiple myeloma (MM). Interferon (IFN)-γ is essential for immune responses, whereas immune checkpoint molecules, such as programmed cell death-1 ligand-1 (PD-L1), mitigate the beneficial anti-tumor immune responses. As HDAC inhibitors alter the immunogenicity and anti-tumor immune responses, we here explored the regulation of PD-L1 expression in MM cells by the clinically available HDAC inhibitor panobinostat in the presence of IFN-γ. IFN-γ activated the STAT1-IRF1 pathway to upregulate PD-L1 expression in MM cells, and panobinostat was able to upregulate their PD-L1 expression without activating the STAT1-IRF1 pathway. Of note, panobinostat enhanced IFN-γR1 expression, which substantially increased the total and phosphorylated levels of STAT1 protein but reduced IRF1 protein levels through proteasomal degradation in the presence of IFN-γ. Panobinostat further enhanced the IFN-γ-mediated durable STAT1 activation in MM cells; *STAT1* gene silencing abolished the PD-L1 upregulation by panobinostat and IFN-γ in combination, indicating a critical role for STAT1. These results suggest that panobinostat enhances PD-L1 expression by facilitating the IFN-γ-STAT1 pathway in a ligand-dependent manner in MM cells with ambient IFN-γ. PD-L1 upregulation should be taken into account when combining immunotherapies with panobinostat.

## INTRODUCTION

Multiple myeloma (MM) progresses while deteriorating immune surveillance. The recent development of immunotherapies with therapeutic monoclonal antibodies has revolutionized the treatment paradigm for MM [1]. The upregulation of CD38 on the surface of MM cells upon treatment with panobinostat has been recently reported [2]; therefore, combinatory treatment of therapeutic anti-CD38 antibodies with panobinostat is expected. Likewise, it is envisioned that induction of antigen editing with histone deacetylase (HDAC) inhibitors can be combined with immunotherapies for MM, including therapeutic antibodies or chimeric antigen receptor (CAR) T cells. In addition, immunomodulatory drugs (IMiDs), such as lenalidomide and pomalidomide, have the potential to activate effector cells, including natural killer (NK) cells, and thus augment antibody-dependent cell-mediated cytotoxicity (ADCC) with therapeutic antibodies [3, 4]. In such current and forthcoming immunotherapies, interferon (IFN)-γ is essential for immune responses [5], whereas immune checkpoint molecules mitigate the beneficial anti-tumor immune responses. Among the immune checkpoint molecules, programmed death 1 (PD-1), a member of the B7 family of cosignaling molecules, and its associated ligand PD-L1 have drawn considerable attention as therapeutic targets in several types of cancers, and inhibitors for the PD-1/PD-L1 axis are often combined with novel anti-cancer agents to maximize their therapeutic efficacy [6–10].

HDAC inhibitors alter the immunogenicity and anti-tumor immune responses [11]. Class I HDAC-specific inhibitors have been demonstrated to upregulate histone acetylation of the *PD-L1* gene promotor to enhance PD-L1 gene expression in melanoma cells [12–14]. In addition, IFN-γ enhances the expression of human leukocyte antigen (HLA) as well as immune checkpoint molecules, including PD-L1, in cancer cells [15]. Thus, cancer cell immunogenicity and anti-tumor immune responses are suggested to be altered by HDAC inhibitors in the presence of activated immune cells producing IFN-γ. Therefore, in the present study, we explored the regulation of PD-L1 expression in MM cells by HDAC inhibitors in the presence of IFN-γ. Panobinostat is a potent pan-HDAC inhibitor that alters gene expression through epigenetic mechanisms, inducing cell cycle arrest and apoptosis in tumor cells. It has been approved in many countries for use in combination with the proteasome inhibitor bortezomib and dexamethasone in relapsed or refractory patients with MM. We demonstrated that panobinostat alone upregulated cytotoxicity-associated molecules, including natural killer group 2D (NKG2D) ligands, UL16-binding protein-2/5/6 (ULBP2/5/6), and MHC class I chain–related proteins A and B (MICA/B) in MM cells in parallel with PD-L1 upregulation. NKG2D receptor is one of the most important activating receptors expressed by NK cells and subsets of T cells in terms of tumor cell recognition and cytotoxicity. NKG2D binds to several different ligands, including ULBPs and MICA/B. ULBP-1, ULBP-2, and ULBP-3 were originally found as ligands for the human cytomegalovirus glycoprotein UL16; up to six different ULBP members have been identified. In the present study, we utilized a monoclonal antibodies specific for MICA/B and ULBP-2/5/6 to examine the expression of NKG2D ligands. Panobinostat further augmented the expression of PD-L1 but not that of NKG2 ligands in MM cells in the presence of IFN-γ. Of note, panobinostat enhanced IFN-γ receptor 1 (IFN-γR1) expression, which markedly increased the total and phosphorylated levels of signal transducer and activator of transcription 1 (STAT1) protein but reduced interferon regulatory factor-1 (IRF1) protein levels via proteasomal degradation in the presence of IFN-γ. These results suggest that panobinostat enhances PD-L1 expression by facilitating the IFN-γ-STAT1 pathway in a ligand-dependent manner in MM cells with ambient IFN-γ. Thus, panobinostat may affect anti-tumor immune responses, and PD-L1 upregulation should be taken into account when combining immunotherapies with panobinostat.

## RESULTS

### IFN-γ increases PD-L1 expression on MM cells via activation of the STAT1-IRF1 pathway

**Figure 1:**
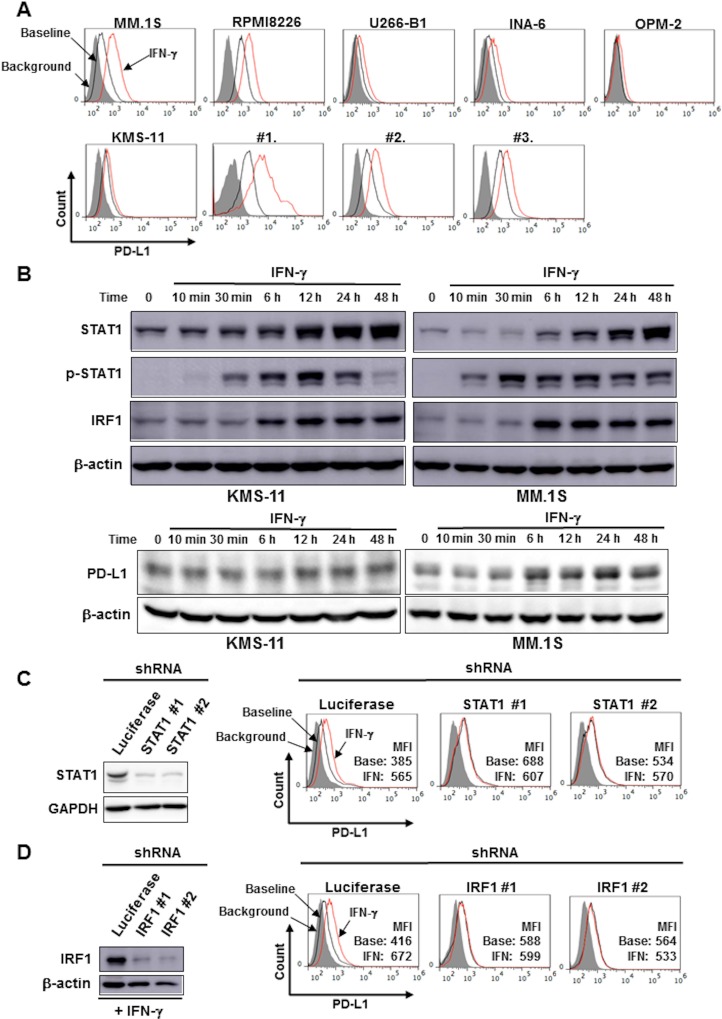
IFN-γ increased PD-L1 expression on MM cells via the STAT1-IRF1 signaling pathway (**A**) Surface expression of PD-L1 on MM cells. MM cell lines as the indicated and primary MM cells (#1, #2, and #3) were cultured in the presence or absence of 100 U/ml of IFN-γ for 24 hours. The surface expression of PD-L1 was then analyzed by flow cytometry. (**B**) Activation of the STAT1-IRF1 pathway. After overnight starvation in culture media containing 1% FBS, KMS-11 and MM.1S cells were incubated in the presence of IFN-γ (100 U/ml) for the indicated time periods. The cells were then harvested, and STAT1, tyrosine-phosphorylated STAT1 (p-STAT1), IRF1 and PD-L1 protein levels were examined by Western blot analysis. β-actin were blotted as loading controls. Effects of *STAT1* (**C**) and *IRF1* (**D**) gene silencing on PD-L1 expression. *STAT1* gene expression was silenced using shRNA in KMS-11 cells. (C) *STAT1* shRNA (clones #1 and #2) or control *Luciferase* shRNA were transfected into KMS-11 cells. The knockdown efficacy was examined by Western blot analysis (left). GAPDH was blotted as loading control. PD-L1 expression on the cells was analyzed by flow cytometry after incubating for 24 hours in the presence or absence of 100 U/ml of IFN-γ. (D) *IRF1* shRNA (clones #1 and #2) or control *Luciferase* shRNA were transfected into KMS-11 cells. The knockdown efficacy was examined by Western blot analysis after incubating for 12 hours in the presence of 100 U/ml of IFN-γ. (left). β-actin were blotted as loading controls. PD-L1 expression on the cells was analyzed by flow cytometry after incubating for 24 hours in the presence or absence of 100 U/ml of IFN-γ. Gray areas indicate background staining with isotype controls. Mean fluorescence intensity (MFI) of PD-L1 is shown. Base, baseline; IFN, IFN-γ.

MM cell lines and primary MM cells expressed PD-L1 on their surface at varying levels (Figure 1A). IFN-γ dose-dependently increased PD-L1 expression on the surface of MM.1S and RPMI8226 cells from 10 to 1000 U/ml (Supplementary Figure 1A). IFN-γ was able to enhance the PD-L1 expression on all MM cells tested (Figure 1A), although extent of the PD-L1 upregulation slightly correlated with its expression levels at baseline.

Treatment with IFN-γ promptly caused STAT1 phosphorylation, followed by the upregulation of STAT1 and IRF1 at protein levels at 6 hours and later in KMS-11 and MM.1S cells (Figure 1B). The STAT1 protein levels markedly increased along with IRF1 protein upon IFN-γ treatment, which is consistent with *STAT1* as well as *IRF1* being STAT1-target genes [16, 17]. These data suggest activation of the STAT1-IRF1 pathway in MM cells by IFN-γ. The knockdown of *STAT1* (Figure 1C) or *IRF1* (Figure 1D) gene marginally affected the basal expression of PD-L1, but was able to abolish the upregulation of PD-L1 on the surface of KMS-11 cells upon treatment with IFN-γ. The activation of the STAT1-IRF1 pathway may play a predominant role in the PD-L1 upregulation by IFN-γ, but not in the basal expression of PD-L1 by MM cells.

### Panobinostat upregulates PD-L1 expression by MM cells without activating the STAT1-IRF1 pathway

**Figure 2:**
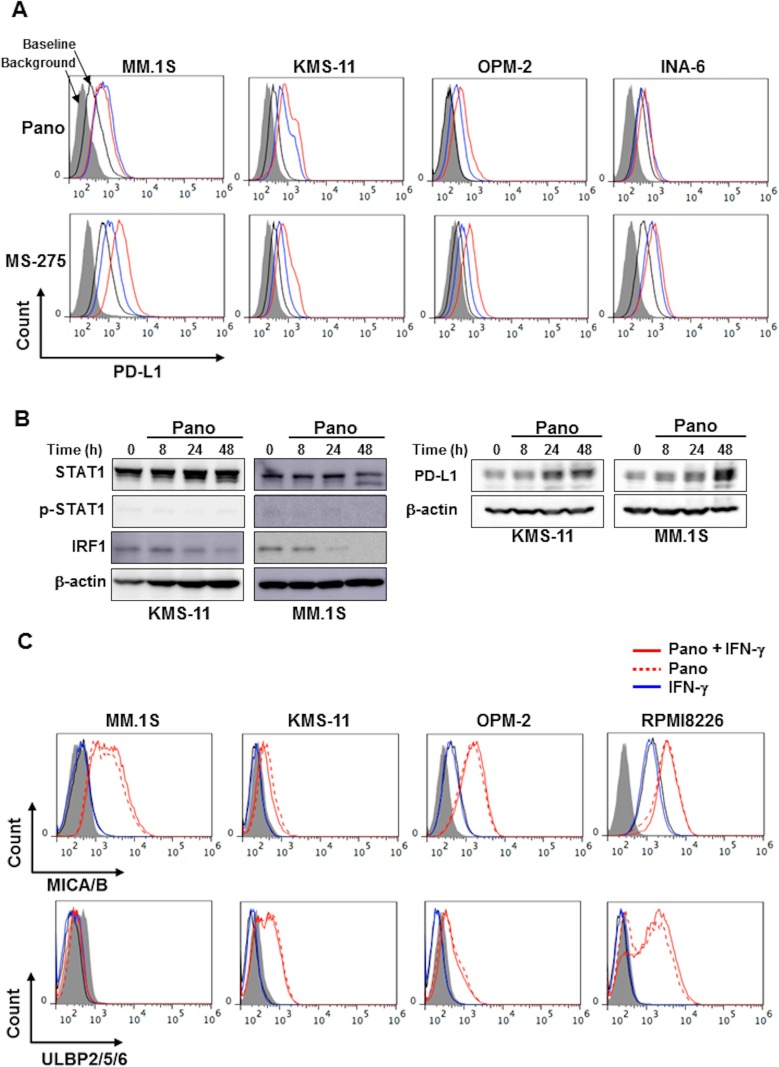
HDAC inhibition upregulates PD-L1 expression in MM cells without activating the STAT1-IRF1 pathway (**A**) Surface expression of PD-L1 on MM cells. MM cell lines as indicated were cultured with either panobinostat at 25 nM (blue) or 100 nM (red) (upper) or MS-275 at 0.25 μM (blue) or 1 μM (red) (lower) for 24 hours. The surface expression of PD-L1 was then analyzed by flow cytometry. (**B**) Analysis of the STAT1-IRF1 pathway. KMS-11 and MM.1S cells were incubated in the presence of panobinostat at 25 nM for the indicated time periods. The cells were then harvested, and STAT1, phosphorylated STAT1 (p-STAT1), IRF1 and PD-L1 protein levels were examined by Western blot analysis. β-actin was blotted as a loading control. (**C**) Surface expression of NKG2D ligands on MM cells. MM cell lines as indicated were cultured with panobinostat at 25 nM in the presence or absence of 100 U/ml of IFN-γ for 24 hours. The surface expression of MICA/B (upper) and ULBP2/5/6 (lower) was then analyzed by flow cytometry. Gray areas indicate background staining with isotype controls. Pano, panobinostat.

The pan-HDAC inhibitor panobinostat and the class I HDAC-specific inhibitor entinostat (MS-275) dose-dependently upregulated PD-L1 expression by MM cells (Figure 2A). In contrast to IFN-γ, treatment with panobinostat only marginally affected the levels of STAT1 protein and its phosphorylation in KMS-11 and MM.1S cells, and reduced their IRF1 protein levels over time (Figure 2B). Therefore, the upregulation of PD-L1 expression by MM cells by panobinostat is likely independent of activation of the STAT1-IRF1 pathway, and may be due to histone acetylation of a *PD-L1* gene promoter as demonstrated in different types of cancer cells [12, 18].

MM cells constitutively express several cytotoxicity-associated molecules, including NKG2D ligands, MICA/B and ULBP2/5/6, on their surface. In contrast to the effects on PD-L1 expression (Figure 1A), IFN-γ marginally affected the expression of MICA/B and ULBP2/5/6 by MM cells (Figure 2C and Supplementary Figure 1B). Of note, panobinostat upregulated ULBP2/5/6 and MICA/B in MM cells irrespective of the concomitant addition of IFN-γ.

### Panobinostat further enhances IFN-γ-mediated durable STAT1 activation and PD-L1 upregulation in MM cells

**Figure 3:**
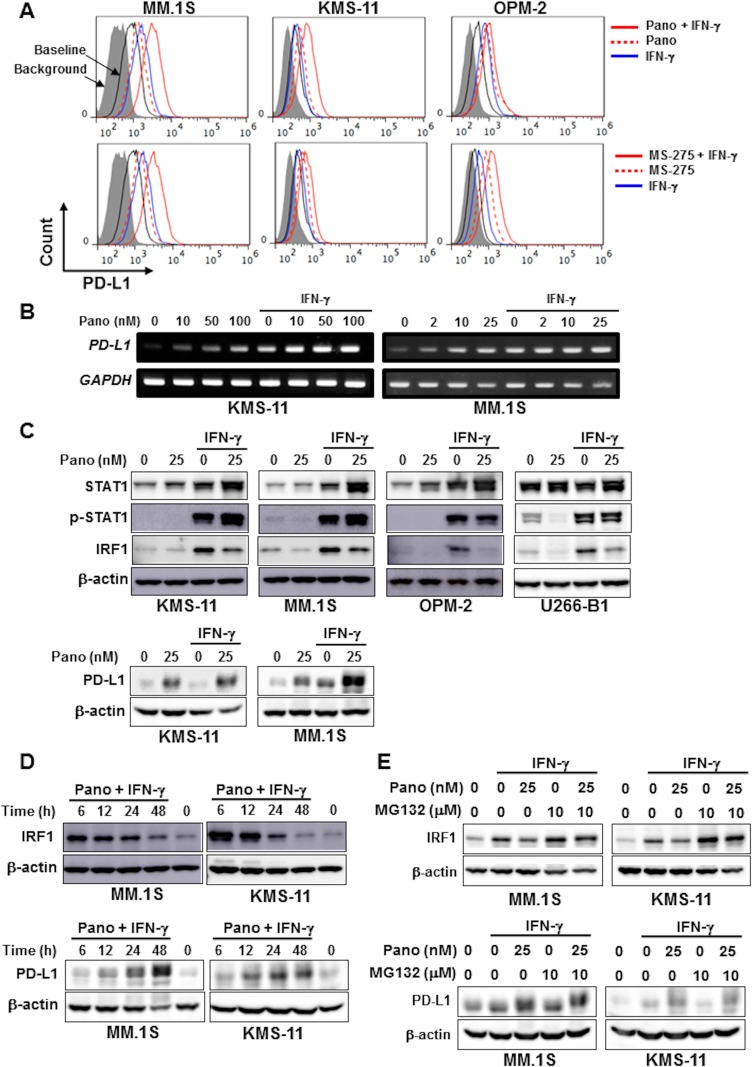
HDAC inhibition upregulates PD-L1 expression by MM cells in combination with IFN-γ (**A**) Surface expression of PD-L1 on MM cells. MM cell lines as indicated were cultured for 24 hours with either 25 nM of panobinostat (Pano) (upper) or 1 μM of MS-275 (lower) in the presence or absence of 100 U/ml of IFN-γ. The surface expression of PD-L1 was then analyzed by flow cytometry. (**B**) *PD-L1* mRNA expression. KMS-11 and MM.1S were cultured for 6 hours with or without panobinostat in the presence or absence of 100 U/ml of IFN-γ. Panobinostat was added at the indicated concentrations. *PD-L1* mRNA expression was analyzed in the MM cells by RT-PCR. *GAPDH* was used as an internal control. (**C**) Analysis of the STAT1-IRF1 pathway. KMS-11 and MM.1S cells were incubated for 24 hours with or without panobinostat at 25 nM in the presence or absence of 100 U/ml of IFN-γ as indicated. The cells were then harvested, and STAT1, phosphorylated STAT1 (p-STAT1), IRF1 and PD-L1 protein levels were examined by Western blot analysis. (**D**) IRF1 protein levels in MM cells. MM.1S and KMS-11 cells were incubated for the indicated time periods in the presence or absence of 25 nM of panobinostat and 100 U/ml of IFN-γ in combination. IRF1 and PD-L1 protein levels were analyzed by Western blot analysis. β-actin was blotted as a loading control. (**E**) IRF1 and PD-L1 protein levels in MM cells. MM.1S cells and KMS-11 cells were cultured for 24 hours with or without panobinostat at 25 nM in the presence or absence of 100 U/ml of IFN-γ as indicated. MG132 was added at 10 μM for the last 4 hours of the incubation period as indicated. Pano, panobinostat.

We next looked at the effects of HDAC inhibition on PD-L1 expression by MM cells in the presence of IFN-γ. Of note, the IFN-γ-induced PD-L1 upregulation in MM cells was further enhanced by panobinostat and MS-275 in MM cell lines (Figure 3A–3C, and Supplementary Figure 1C). The cooperative PD-L1 upregulation by panobinostat and IFN-γ was further confirmed in primary MM cells (Supplementary Figure 1D). In contrast to the marginal effects of IFN-γ on the upregulation of ULBP2/5/6 and MICA/B expression by HDAC inhibition, PD-L1 upregulation by these HDAC inhibitors was further enhanced in MM cells in the presence of IFN-γ.

Although panobinostat alone did not activate the STAT1-IRF1 pathway in MM cells (Figure 2B), panobinostat further enhanced the total and phosphorylated levels of STAT1 protein but reduced IRF1 protein levels in MM cells in the presence of IFN-γ (Figure 3C). Furthermore, IRF1 protein levels decreased over time after treatment with panobinostat and IFN-γ in combination (Figure 3D), although IFN-γ alone continuously increased IRF1 protein levels over 48 hours in these MM cells (Figure 1B). The upregulation of PD-L1 on the surface of MM cells upon treatment with panobinostat and IFN-γ in combination remained at day 2 (Supplementary Figure 1E), when IRF1 protein levels decreased in MM cells (Figure 3D), suggesting limited contribution of IRF1 to the sustained PD-L1 upregulation by HDAC inhibition in the presence of IFN-γ. In addition, the upregulation of PD-L1, MICA/B, and ULBP2/5/6 on MM cells was limited or marginal in some MM cells by IFN-γ and/or panobinostat at 8 hours compared to 24 hours (Supplementary Figure 1F). However, *IRF1* mRNA expression was markedly increased in the MM cells by IFN-γ, which remained high after the further addition of panobinostat (Supplementary Figure 2A). Of note, the proteasome inhibitor MG132 further increased IRF1 protein levels upregulated by IFN-γ in MM.1S and KMS-11 cells even in the presence of panobinostat (Figure 3E), suggesting proteasomal degradation of IRF1 protein. MS-275 similarly increased the protein levels of STAT1 and phosphorylated STAT1 in MM.1S and KMS11 cells in the presence of IFN-γ (Supplementary Figure 2B), and the HDAC6 inhibitor ACY-1215 also upregulated PD-L1 expression on these MM cells (Supplementary Figure 2C). These results demonstrate that panobinostat can enhance IFN-γ-mediated stable STAT1 activation and PD-L1 upregulation in MM cells while mitigating IRF1 protein levels over time.

### Panobinostat markedly increases STAT1 levels in MM cells in the presence of IFN-γ

**Figure 4:**
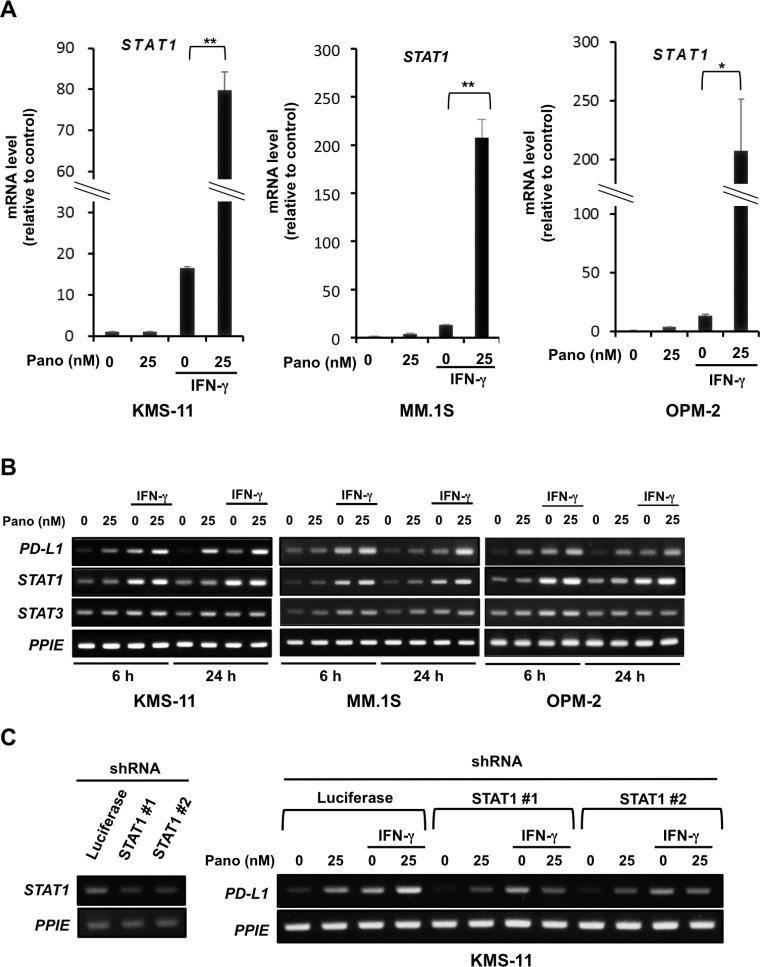
STAT1 upregulation in MM cells by panobinostat in the presence of IFN-γ (**A**) *STAT1* mRNA expression in MM cells. KMS-11, MM.1S and OPM-2 cells were cultured in triplicate for 24 hours with or without 25 nM of panobinostat in the presence or absence of 100 U/ml of IFN-γ. *STAT1* mRNA expression was quantified by quantitative RT-PCR. Ratios of *STAT1* over *PPIE* mRNA levels were calculated for a normalized target value (defined as 1). *PPIE* was used as an internal control. Results were expressed as the mean ± SD. ^*^p < 0.05, ^**^p < 0.01. (**B**) *STAT1* and *STAT3* expression in MM cells. KMS-11, MM.1S and OPM-2 cells were cultured for 6 or 24 hours with or without 25 nM of panobinostat in the presence or absence of 100 U/ml of IFN-γ. *STAT1*, *STAT3* and *PD-L1* mRNA expression was analyzed in the MM cells by RT-PCR. (**C**) Effects of *STAT1* gene silencing on PD-L1 expression. *STAT1* shRNA (clones #1 and #2) or control *Luciferase* shRNA were transfected into KMS-11 cells. The knockdown efficacy was examined by RT-PCR (left). The cells were cultured for 6 hours with or without 25 nM of panobinostat in the presence or absence of 100 U/ml of IFN-γ. *PD-L1* mRNA expression was analyzed by RT-PCR. *PPIE* was used as an internal control. Pano, panobinostat.

As panobinostat mitigated IRF1 protein levels in MM cells in the presence of IFN-γ over time (Figure 3D), and because *PD-L1* is a target gene of STAT1 as well as IRF1 [19–22], we further clarified the role of STAT1 in the upregulation of PD-L1 in MM cells by panobinostat and IFN-γ in combination. Real-time RT-PCR revealed that IFN-γ alone was able to increase *STAT1* mRNA expression in MM cells (Figure 4A). Of note, panobinostat in combination with IFN-γ further increased *STAT1* mRNA expression in MM cells 5- to over 10-times greater than that by IFN-γ alone at 24 hours. *STAT3* mRNA expression was weakly increased in MM cells upon treatment with panobinostat and IFN-γ in combination (Figure 4B). In real-time RT-PCR, the increase of *STAT3* mRNA was much less than that of *STAT1* mRNA in MM cells especially upon treatment with panobinostat and IFN-γ in combination (Supplementary Figure 3A). *STAT1* gene silencing by shRNA mostly abolished the enhancement of PD-L1 expression at mRNA and protein levels in MM cells by panobinostat in the presence of IFN-γ at 24 hours (Figure 4C and Supplementary Figure 3B), although panobinostat alone was able to induce PD-L1 expression largely through histone acetylation of the *PD-L1* gene promotor in the absence of IFN-γ. As IRF1 protein levels decreased in MM cells by panobinostat in the presence of IFN-γ at 24 hours (Figure 3C and 3D), STAT1 upregulation and activation likely play an important role in the cooperative and stable upregulation of PD-L1 in MM cells by panobinostat and IFN-γ in combination.

### Panobinostat upregulates IFN-γR1 expression in MM cells

**Figure 5:**
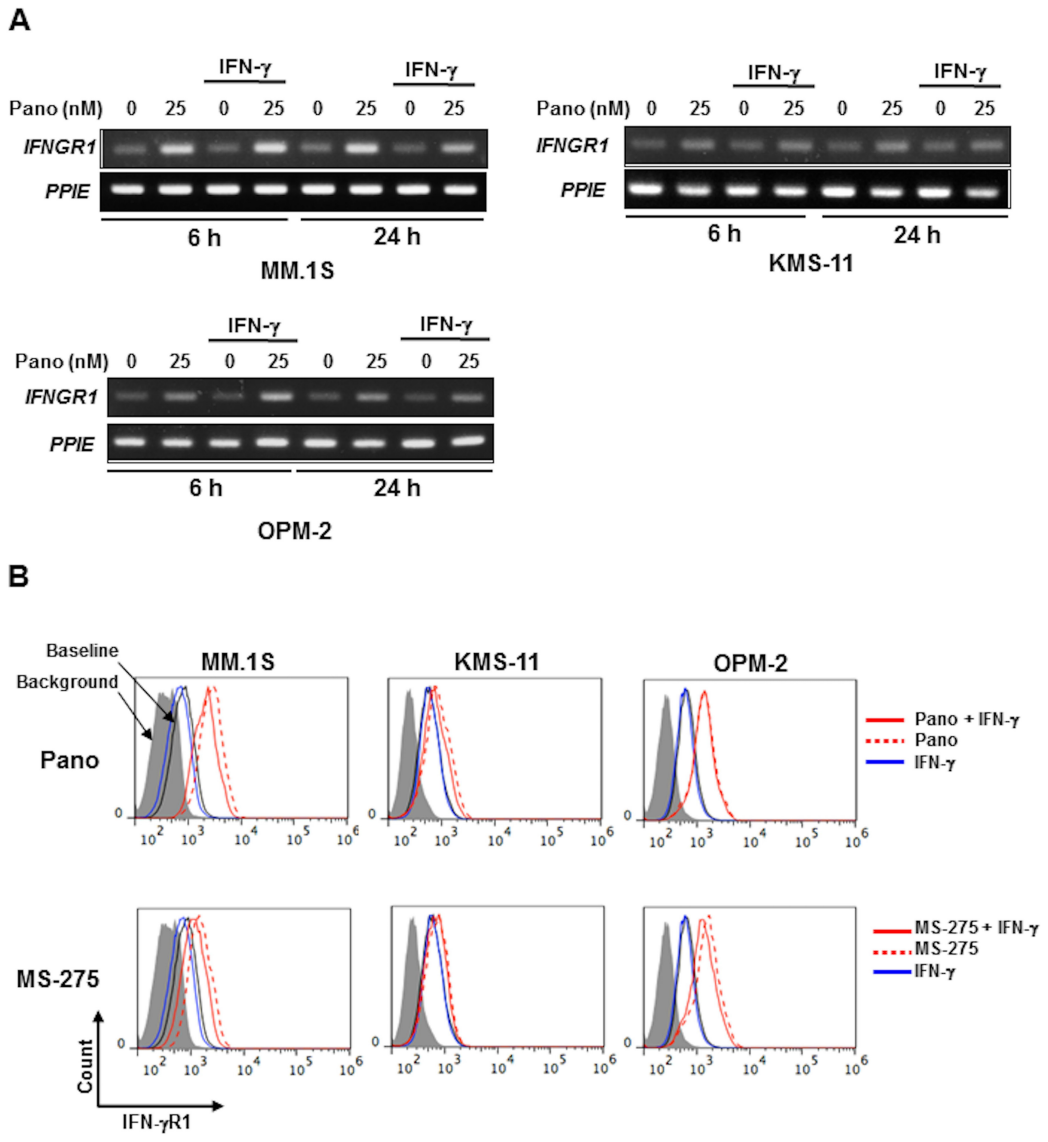
HDAC inhibition upregulates IFN-γR1 expression in MM cells (A) *IFNGR1* expression in MM cells. KMS-11, MM.1S and OPM-2 cells were cultured for 6 or 24 hours with or without 25 nM of panobinostat in the presence or absence of 100 U/ml of IFN-γ. *IFNGR1* mRNA expression was analyzed in the MM cells by RT-PCR. *PPIE* was used as an internal control. (B) Surface expression of IFN-γR1 on MM cells. KMS-11, MM.1S and OPM-2 cells were cultured for 24 hours with or without 25 nM of panobinostat (upper) or 1 μM of MS-275 (lower) in the presence or absence of 100 U/ml of IFN-γ. The surface expression of IFN-γR1 was analyzed by flow cytometry. Pano, panobinostat.

Although the upregulation of PD-L1 by panobinostat alone appears to be independent of the activation of the STAT1-IRF1 pathway in MM cells, panobinostat was able to greatly increase STAT1 protein as well as its phosphorylation levels in combination with IFN-γ. To clarify the underlying mechanisms for the activation of STAT1 by panobinostat in the presence of IFN-γ, we examined the expression of IFN-γR1, a receptor for IFN-γ, in MM cells. Panobinostat upregulated *IFNGR1* mRNA expression in MM cells irrespective of the addition of IFN-γ (Figure 5A). The expression of IFN-γR1 was indeed increased on the surface of the MM cells by panobinostat, as well as MS-275, even in the presence of IFN-γ (Figure 5B and Supplementary Figure 4A). These results suggest that panobinostat facilitates activation of the IFN-γ-STAT1 pathway in a ligand-dependent manner in MM cells.

### Lenalidomide and pomalidomide enhance PD-L1 expression on MM cells in the presence of IFN-γ

**Figure 6:**
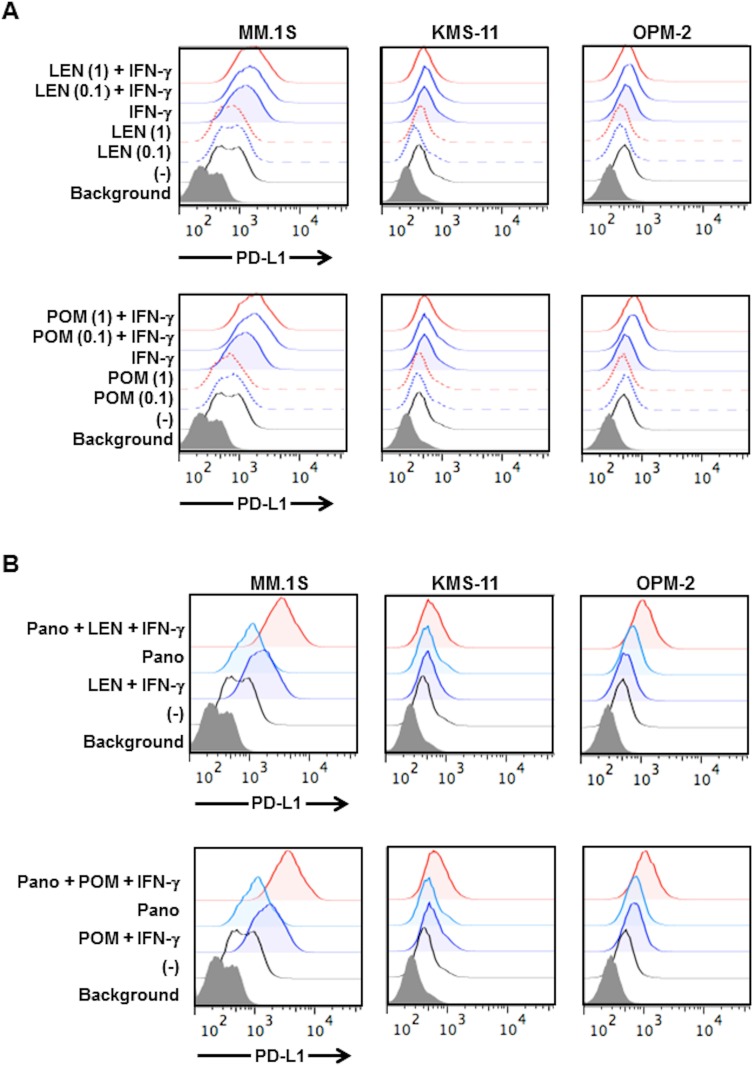
Effects of lenalidomide and pomalidomide on PD-L1 expression on MM cells (**A**) PD-L1 expression on MM cells by lenalidomide and pomalidomide. KMS-11, MM.1S and OPM-2 cells were cultured for 24 hours with or without lenalidomide or pomalidomide in the presence or absence of 100 U/ml of IFN-γ. Lenalidomide or pomalidomide were added at 1.0 or 0.1 μM, respectively. LEN, lenalidomide, POM, pomalidomide. (**B**) Effects of lenalidomide and pomalidomide on PD-L1 upregulation on MM cells by panobinostat. KMS-11, MM.1S and OPM-2 cells were cultured for 24 hours with or without 1 μM of lenalidomide or 0.1 μM of pomalidomide in the presence or absence of 100 U/ml of IFN-γ. Panobinostat was further added at 25 nM as indicated. The surface expression of PD-L1 was analyzed by flow cytometry. Pano, panobinostat.

Second generation IMiDs, namely lenalidomide and pomalidomide, have drawn considerable attention for their activation of effector cells with IFN-γ production, including NK cells and cytotoxic T cells in patients receiving these IMiDs [23]. Lenalidomide has been reported to reduce PD-L1 expression on RPMI8226 and primary MM cells [24]. However, under our experimental conditions, lenalidomide did not reduce PD-L1 expression in MM cell lines (Figure 6A). On the other hand, treatment with lenalidomide enabled the upregulation of PD-L1 expression on the surface of MM cells by IFN-γ, or rather enhanced PD-L1 expression in combination with IFN-γ (Figure 6A). Similar results were obtained with pomalidomide (Figure 6A). The addition of panobinostat further enhanced PD-L1 expression on the surface of MM cells in the presence of lenalidomide or pomalidomide in combination with IFN-γ (Figure 6B). Panobinostat impaired MM cell viability; however, IFN-γ did not further affect and lenalidomide or pomalidomide marginally or only weakly suppressed the viability of the MM cells in combination with panobinostat (Supplementary Figure 4B). However, lenalidomide and pomalidomide did not enhance the IFN-γ-STAT1 pathway in MM cells (Supplementary Figure 4C). The underlying mechanism for PD-L1 induction by the combination treatment with IMiDs and IFN-γ remains to be clarified. These results suggest that lenalidomide and pomalidomide do not to negatively impact PD-L1 expression on MM cells, and that PD-L1 expression on MM cells may be enhanced with ambient IFN-γ elaborated by immune cells in patients receiving these IMiDs.

## DISCUSSION

PD-1 and its associated ligand PD-L1 play a key role in downregulating anti-tumor immune responses. The PD-1/PD-L1 axis has been reported as a master immune checkpoint in MM cells [25]. From the present results, the PD-1/PD-L1 axis may function in immune tolerance to treatment with HDAC inhibitors especially with ambient IFN-γ, whereas HDAC inhibitors directly impair MM cell viability.

As generally accepted for immune cells and other types of cancer cells [26–28], IFN-γ activated the STAT1-IRF1 pathway, and thereby upregulated PD-L1 expression in MM cells (Figure 1A and 1B). On the other hand, panobinostat alone did not activate the STAT1-IRF1 pathway in MM cells in the absence of IFN-γ (Figure 2B), but enhanced *PD-L1* gene expression via histone acetylation. Of note, panobinostat markedly enhanced *STAT1* gene expression in parallel with PD-L1 upregulation in MM cells in the presence of IFN-γ (Figure 4A). To clarify the mechanism by which panobinostat enhances and activates STAT1 in the presence of IFN-γ, we looked at the expression of IFN-γR1, a receptor for IFN-γ, in MM cells. Panobinostat upregulated IFN-γR1 on the surface of MM cells, which facilitates the IFN-γ-STAT1 pathway in MM cells. Because panobinostat increased IFN-γR1 expression in MM cells at mRNA as well as protein levels, panobinostat is suggested to at least in part enhance transcription of *IFN-γR1* gene through its HDAC inhibitory activity. Precise mechanisms for IFN-γR1 upregulation in MM cells by panobinostat remain to be clarified.

IFN-γ binds to its receptor IFN-γR1 to trigger downstream IFN-γ signaling to phosphorylate STAT1 and thereby enhance the transcription of *STAT1* gene. Silencing of *IFNGR1* gene by shRNA abolished the PD-L1 upregulation in MM cells by IFN-γ in the presence or absence of panobinostat, indicating a critical role of IFN-γR1 in mediating intracellular IFN-γ signaling (Supplementary Figure 5B). IFN-γR1 upregulation in MM cells by panobinostat can allow larger amounts of exogenous IFN-γ to bind to IFN-γR1, and thus facilitate the IFN-γ-mediated signaling pathways in a ligand-dependent manner. IFN-γ can stimulate various signaling pathways, including NF-κB-mediated ones, which may further enhance the STAT1 expression. Precise mechanisms remain to be addressed with specific inhibition of various signaling pathways activated by IFN-γ. As *STAT1* is a target of STAT1 itself [29], transcription of the *STAT1* gene triggered by IFN-γ may auto-amplify STAT1 levels in MM cells, which are further enhanced by HDAC inhibition.

*STAT1* gene silencing with shRNA almost completely abolished the upregulation of *PD-L1* expression in KMS-11 cells by IFN-γ (Figure 4C), indicating the predominant role of STAT1 in the PD-L1 upregulation. Although PD-L1 expression is upregulated through activation of the STAT1-IRF1 pathway by IFN-γ (Figure 1), panobinostat mitigated the IRF1 protein levels in MM cells upregulated by IFN-γ over time (Figure 3D). *PD-L1* is a target gene of STAT1 in other types of malignant cells [22, 30, 31]; therefore the augmentation of IFN-γ-induced PD-L1 upregulation in MM cells by panobinostat may be largely due to transcription of STAT1 rather than due to IRF1.

Panobinostat reduced IRF1 protein levels in MM cells in the presence of IFN-γ at 24 hours (Figure 3C and 3D), whereas IRF1 mRNA levels remained at increased levels (Supplementary Figure 2A). However, the IRF1 protein levels were restored in MM cells by proteasome inhibition even in the presence of panobinostat (Figure 3E). IRF1 is a client protein of the molecular chaperone heat shock protein 90 (Hsp90) and is protected from proteasomal degradation by Hsp90 [32]. Acetylation of Hsp90 is required for its chaperone activity [33], and HDAC inhibition has been demonstrated to induce proteasomal degradation of IRF1 protein [34, 35]. Consistently, proteasome inhibition restored IRF1 protein levels in MM cells which were suppressed by panobinostat in the presence of IFN-γ (Figure 3E). Therefore, IRF1 protein is suggested to be subject to protein degradation in MM cells by panobinostat. In addition, treatment with the Hsp90 inhibitor 17-allylamino-demothoxy geldanamycin (17-AAG) inhibited substantially reduced IRF1 protein levels in MM cells in the presence or absence of IFN-γ (Supplementary Figure 5A), indicating HSP90 as a main factor. Because Hsp90 chaperone activity is known to be regulated by reversible acetylation and controlled by the deacetylase HDAC6 [36, 37], panobinostat, a pan-HDAC inhibitor, is suggested to suppress Hsp90 chaperone activity through inhibition of HDAC6. These results warrant further study on the precise role of HDAC6 in the IFN-γ signaling pathway through modulation of Hsp90 chaperone activity and IRF1 stability in MM cells. The precise mechanisms by which panobinostat reduces IRF1 protein in MM cells remain to be further clarified.

In parallel with PD-L1 upregulation, panobinostat directly induced cytotoxicity-associated molecules, including NKG2 ligands, ULBP2/5/6 and MICA/B, in MM cells (Figure 2C). Panobinostat further augmented the expression of PD-L1 but not that of these NKG2 ligands in MM cells in the presence of IFN-γ. HDAC inhibitors can regulate the transcription of a variety of immune-stimulating as well as immune-suppressing genes, and can modulate the activity of immune effector and suppressor cells; and thus, HDAC inhibitors have ambivalent immunomodulatory activity. Besides their immunomodulatory activity, the benefit conferred by HDAC inhibitors may be contingent upon their ability to enhance the expression of antigens of tumor cells targeted by therapeutic monoclonal antibodies, including the anti-CD38 antibody daratumumab. Indeed, panobinostat has been reported to upregulate CD38 expression in MM cell lines and primary MM samples, which augments the *in vitro* cytotoxic effects of daratumumab on MM cells [2]. We demonstrated that HDAC inhibitors, including panobinostat, upregulate NKG2 ligands (ULBP2/5/6 and MICA/B) on MM cells. Therefore, HDAC inhibitors can be expected to promote anti-tumor immunity in the context of immune cell activation with the therapeutic anti-CD38 antibody daratumumab. However, in parallel with the upregulation of the NKG2 ligands, the expression of PD-L1 on MM cells was found to be upregulated by panobinostat, which is further enhanced in combination with IFN-γ, although IFN-γ production is increased by ambient immune cells activated by immunotherapies in a tumor microenvironment. The present study provides a rationale for potential combinatory treatment with inhibitors of the PD-L1/PD-1 immune checkpoint to effectively elicit immunogenic actions of HDAC inhibition.

STAT3 protein and its phosphorylation levels are increased in MM cells to mediate many cellular functions responsible for MM cell growth and survival [21, 38]. In contrast to the pronounced upregulation of *STAT1* gene expression (Figure 4A), *STAT3* gene expression was much less increased in MM cells upon treatment with panobinostat and IFN-γ in combination (Supplementary Figure 3A). In cancer cells, STAT1 and STAT3 may interfere with each other, and perturbation of the balance of STAT1 and STAT3 levels is suggested as a novel therapeutic strategy for cancers [39]. In addition, because unphosphorylated STAT1 can also act as a transcription factor for several genes [16], the sustainable increase of STAT1 protein by panobinostat in the presence of IFN-γ may affect MM cell biology. The biological roles of STAT1 accumulation in MM cells by panobinostat and IFN-γ in combination should be further clarified.

Lenalidomide and pomalidomide are able to exert therapeutically beneficial effects with immune activation [3, 40, 41]. In the present study, these IMiDs slightly upregulated PD-L1 in MM cells in the presence of IFN-γ. In responders to IMiDs, IMiDs are able to stimulate immune effector cells to enhance IFN-γ production in an ambient tumor microenvironment, which can stimulate the IFN-γ-STAT1 pathway in MM cells. There may be a mechanism of PD-L1 upregulation in MM cells at least in part by IFN-γ produced from activated immune effector cells in responders to IMiDs. The impact of PD-L1 upregulation on MM cells with ambient IFN-γ should be clarified in patients with MM receiving these IMiDs, especially in combination with therapeutic antibodies for augmentation of ADCC activity.

We summarized the findings in the present study in Supplementary Figure 6. Multiple signaling pathways can regulate PD-L1 expression in tumor cells and the microenvironmental cells surrounding them [9, 10, 25]. According to the present study, HDAC inhibitors should be considered to curb PD-L1 upregulation in combination with potent immune therapies. Although immune therapies with novel agents open new avenues for cancer treatment, we need to clarify the status of PD-L1 expression under the immune therapies and consider implementing immune checkpoint inhibition during treatment.

## MATERIALS AND METHODS

### Reagents

The following reagents were purchased from the indicated manufacturers: panobinostat (LBH589) and ACY-1215 from Cayman Chemical Company (MN, USA); entinostat (MS-275) from Selleck (Houston, USA); lenalidomide from Santa Cruz Biotechnology (CA, USA); pomalidomide from Toronto Research Chemicals (Toronto, Canada); recombinant human (rh) IFN-γ from R&D systems (MN, USA); rh IL-6 from Pepro Tech Inc. (London, UK) and MG132 from Selleck (Houston, USA); phycoerythrin (PE)-conjugated monoclonal antibodies (mAbs) against human PD-L1 (#329706), human MICA/MICB (#320906), human CD119 (IFN-γR1) (#308606) and mouse IgG_1_-κ isotype control (#400114) from BioLegend (CA, USA); human ULBP-2/5/6 (FAB1298P) from R&D systems (MN, USA); fluorescein isothiocyanate (FITC)-conjugated mAbs against human CD38 (#555459) from BD Biosciences (CA, USA); anti-phosho-STAT1 (Tyr701) antibody (#7649), anti-STAT1 antibody (#14995), anti-PD-L1 antibody (#13684) and anti-GAPDH antibody (#5174) from Cell Signaling; anti-IRF1 antibody (sc-497) from Santa Cruz (CA, USA); and anti-β-actin antibody (A5316) from Sigma-Aldrich (St. Louis, MO, USA); 17-AAG from Calbiochem EMD Biosciences, Inc. (La Jolla, CA, USA).

### Cells and cultures

The human MM cell lines RPMI8226, U266-B1 and KMS-11 were obtained from the American Type Culture Collection (ATCC, Rockville, MD); OPM-2 was purchased from the German Collection of Microorganisms and Cell Cultures (Braunschweig, Germany). INA-6 and MM.1S were kindly provided by Dr. Renate Burger (University of Kiel, Kiel, Germany) and Dr. Steven Rosen (Northwestern University, Chicago, IL, USA), respectively. Cells were cultured in RPMI1640 medium (Sigma-Aldrich, St. Louis, MO, USA) supplemented with 5% fetal bovine serum (FBS), 100 U/ml of penicillin G and 100 μg/ml of streptomycin at 37°C in 5% CO_2_. INA-6 was cultured in growth media containing 10% FBS and 10 ng/ml of IL-6. Bone marrow mononuclear cells (BMMCs) were separated by Ficoll centrifugation (Ficoll-Paque PLUS, GE Healthcare Japan Corporation) as described previously [42]. MM cells were purified from BMMCs using anti-CD138 microbeads and the Miltenyi magnetic cell sorting system (Miltenyi Biotec, Auburn, CA, USA) according to the manufacturer's instructions. All procedures involving human specimens were performed with written informed consent according to the Declaration of Helsinki using a protocol approved by the Institutional Review Board for human protection.

### Flow cytometry

Approximately 5 × 10^5^ cells were washed with PBS containing 1% BSA and stained with antibodies or the isotype control for 30 minutes on ice as described previously [43, 44]. After washing, cells were analyzed with Gallios (BECKMAN COULTER, CA, USA). Data were edited using FlowJo software (BD Biosciences San Jose, CA, USA).

### Immunoblotting

Cells were washed with PBS, and then lysed using RIPA buffer containing protease inhibitors (Sigma-Aldrich, MO, USA), phenylmethylsulfonyl fluoride, phosphatase inhibitors and dithiothreitol (Wako, Osaka, Japan). The protein concentration was measured using the DC protein assay (Bio-Rad, CA, USA). Cell lysates and conditioned media were electrophoresed on 10% SDS-PAGE gels and blotted onto polyvinylidene difluoride membranes (Millipore, Bedford, MA, USA). After blocking with 3% bovine serum albumin (Wako, Osaka, Japan) or 5% non-fat dry milk for 1 hour and washing 3 times with 1 × TBS containing 0.1% Tween (MP biomedicals, Illkirch, France) (TBST), the membranes were incubated with primary antibodies overnight at 4°C. The membranes were washed 3 times with TBST and incubated with a horseradish-conjugated secondary antibody for 1 hour at room temperature. After incubation, the membranes were washed 3 times with TBST and incubated with an enhanced chemiluminescence reagent (Millipore, MA, USA) for 5 minutes.

### RNA extraction, reverse-transcription polymerase chain reaction (RT-PCR) and quantitative real-time PCR (qRT-PCR)

TRI REAGENT (Cosmo Bio co., LTD, Tokyo, Japan) was used to isolate RNA. Reverse transcription for complementary DNA (cDNA) synthesis was carried out using PrimeScript RT Master Mix (Takara Bio Inc., Shiga, Japan). One-tenth of the cDNA template was used for subsequent PCR analysis in a 20-μl reaction solution using TaKaRa Ex Taq Hot Start Version (Takara Bio Inc.) with 23 to 35 cycles of 95°C for 30 seconds, 55 to 60°C for 30 seconds, and 72°C for 30 seconds. qRT-PCR was performed in a 96-well plate using the standard curve method and 7300 Real Time PCR system (Applied Biosystems, CA, USA). The reaction contained Power SYBR Green Master Mix (Thermo Fisher Scientific, IL, USA), reverse primer, forward primer, template cDNA, and nuclease-free water. The total volume was 20 μl/reaction. The protocol was as follows: holding stage was 95°C for 10 minutes and the cycling stage was 40 cycles at 95°C for 15 seconds and 60°C for 60 seconds. The following primers were used: *PD-L1* forward 5′-TGACCAGCACACTGAGAATCAA-3′ and *PD-L1* reverse 5′- TGGAGGATGTGCCAGAG GT-3′; *IRF1* forward 5′-CAAATCCCGGGGCTCATCTG-3′ and *IRF1* reverse 5′-CTGCTTTGTATCGGCCTGTGTG-3′; *STAT1* forward 5′-TGGTGAAATTGCAAGAGCTGA-3′ and *STAT1* reverse 5′-GTGTGCGTGCCCAAAATG-3′; *STAT3* forward 5′-AGCTGGCTGACTGGAAGAGG-3′ and *STAT3* reverse 5′-TTGTTGACGGGTCTGAAGTT G-3′; *IFNGR1* forward 5′-GTTAAAGCCAGGGTTGG ACA-3′ and *IFNGR1* reverse 5′-ATCGACTTCCTGCTC GTCTC-3′ [45]; glyceraldehyde-3-phosphate dehydrogenase (*GAPDH*) forward 5′-TGTCTTCACCACCATGGAGAAGG-3′ and *GAPDH* reverse 5′-GTGGATGCAGGGATGATG TTCTG-3′; peptidylprolyl isomerase E (*PPIE*) forward 5′- TGGACGTACAATTCGTGTCAA-3′ and *PPIE* reverse 5′- GGCTCTGACCCTTCTTCCTC-3′.

### Short hairpin RNA (shRNA) transfection

Lentiviral production and infection were performed as described previously [46]. 293T cells at a density of 3 × 10^5^ were seeded in 6-well plates with DMEM containing 10% FBS and incubated for 24–30 hours. The shRNA-encoding transfer vector (500 ng/well), dvpr (500 ng/well) and VSV-G (50 ng/well) were mixed with TransIT-LT1 transfection reagent-containing OPTI-MEM media (Thermo Fisher Scientific, IL, USA). After a 30 minute incubation at room temperature, 293T cells were transfected with 3 different plasmids for 18 hours at 37°C in 5% CO_2_. After transfection, fresh media was added. Twenty-four hours later, virus particles were harvested. KMS-11 cells were transfected with Mission Lentiviral transduction particles containing *STAT1* shRNA (NM_016166 clone TRCN0000257327 and TRCN0000231899, Sigma-Aldrich) or *IRF1* shRNA (NM_002198 clone TRCN0000218571 and TRCN0000229658, Sigma-Aldrich) or *IFNGR1* shRNA (NM_000416 clone TRCN0000304196) in the presence of polybrene (8 mg/ml). After incubation for 3–5 hours in 6-well plates, cells were washed with PBS and fresh growth media was added. Cells were selected with puromycin (1 μg/ml) for 48 hours. Luciferase was used as the control. The efficacies of inhibition by shRNA was evaluated by immunoblotting.

### Cell viability assay

Cells were plated out in triplicate on 96-well culture plates and incubated with drugs. The number of viable cells was determined by the Cell Counting Kit-8 (WST-8) assay (DOJINDO, Kumamoto, Japan) according to the manufacturer's instructions. The absorbance of each well was measured at 450 nm with a microplate reader (Model 450 micro plate reader; Bio-Rad Laboratories, Hercules, CA, USA).

### Statistical analysis

Significance was determined by the paired *t*-test. A value of *P* < 0.05 was considered significant.

## SUPPLEMENTARY MATERIALS FIGURES


