# Supplementary material for: PD-L1 upregulation in myeloma cells by panobinostat in combination with interferon-γ
Source: Oncotarget. 2019 Mar 8;10(20):1903–17. doi: 10.18632/oncotarget.26726 (PMC6443002; doi:10.18632/oncotarget.26726)
Supplement: Supplementary file 1 [file oncotarget-10-1903-s001.pdf]

PD-L1 upregulation in myeloma cells by panobinostat in combination with interferon-γ

SUPPLEMENTARY MATERIALS

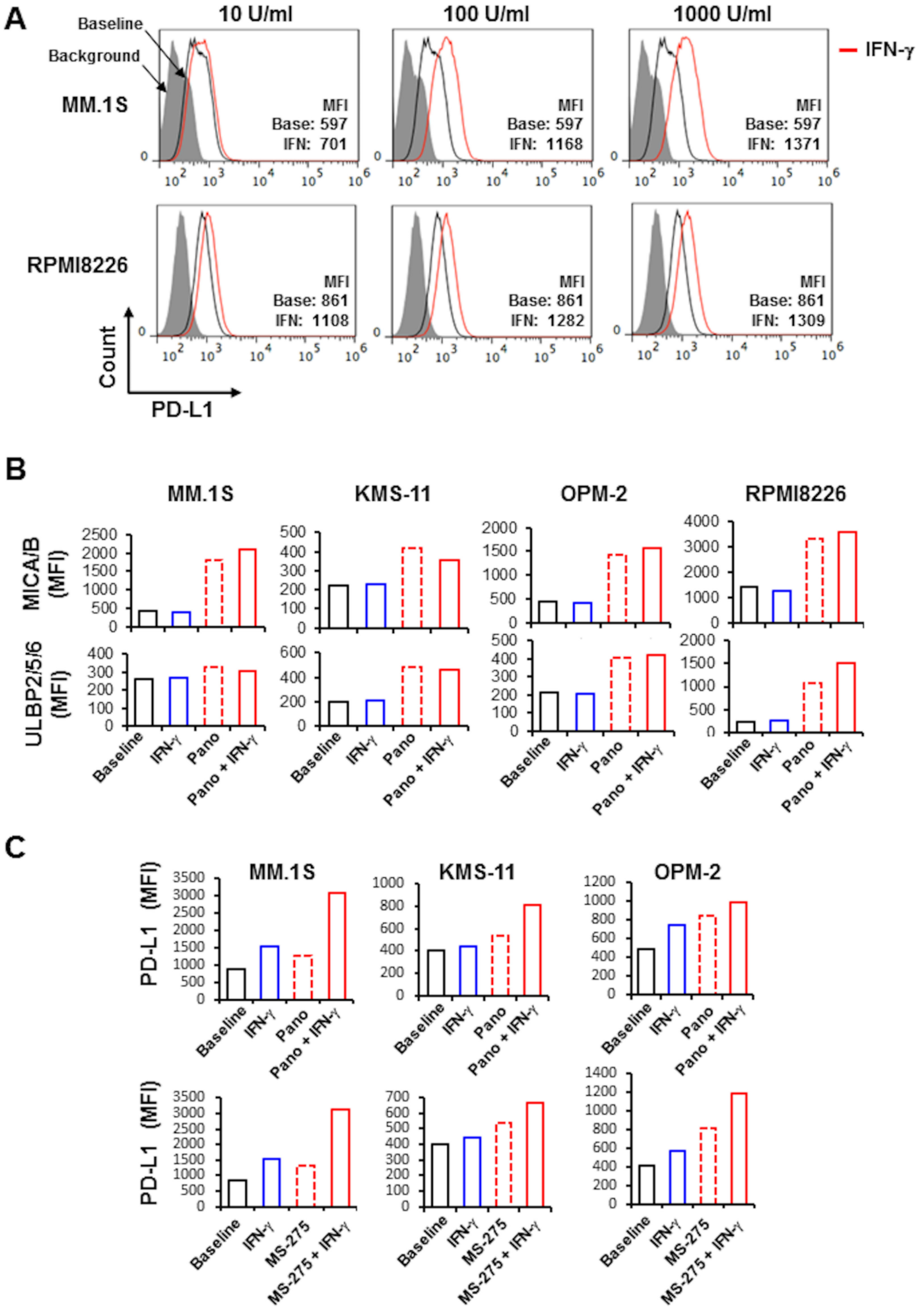

D

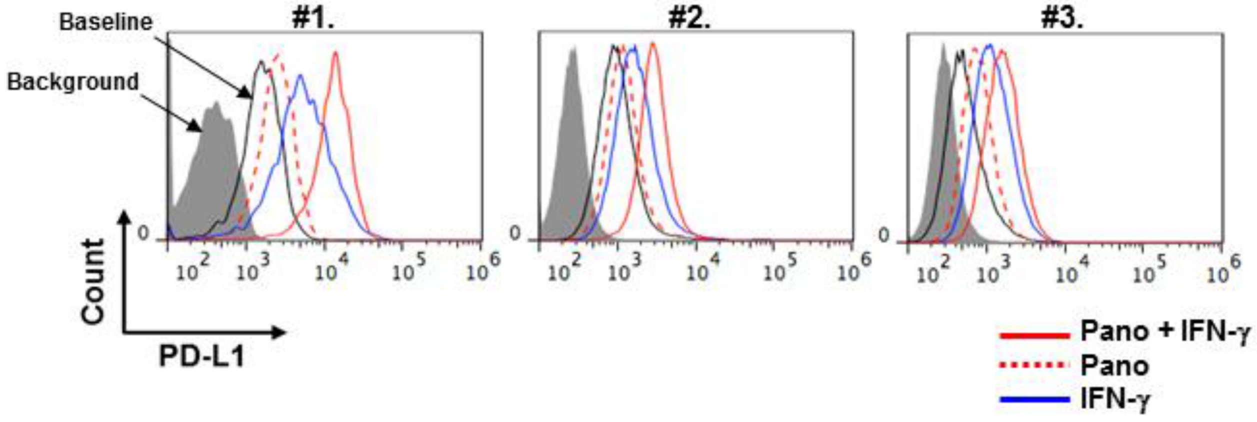
#1.
#2.
#3.
Baseline
Background
0
10^2
10^3
10^4
10^5
10^6
Count
PD-L1
Pano + IFN-γ
Pano
IFN-γ

E

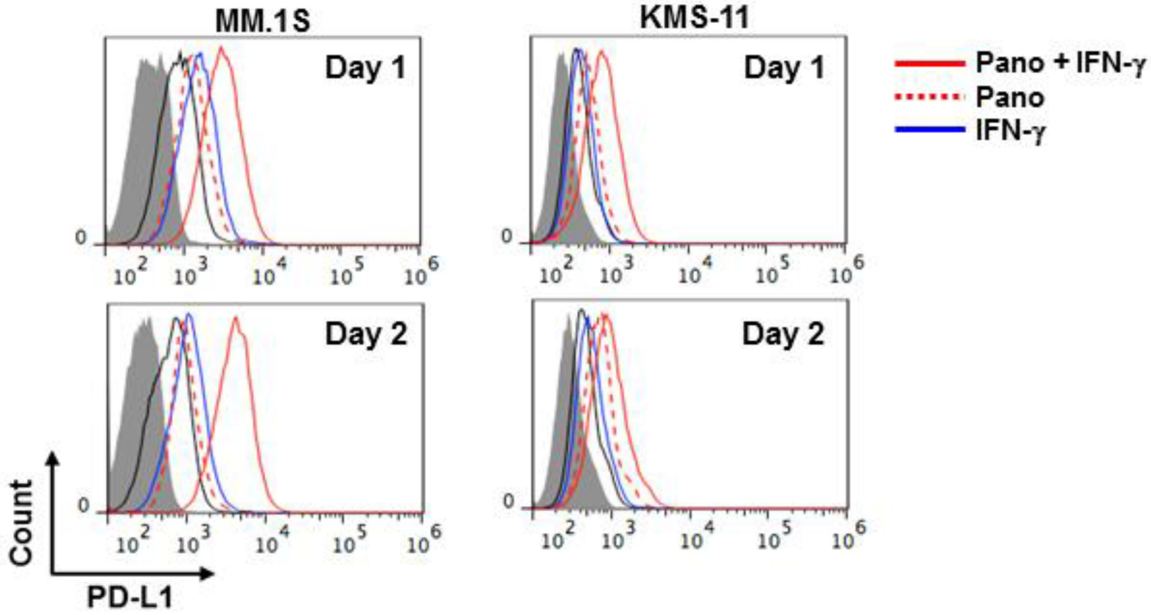
MM.1S
KMS-11
Day 1
Day 2
0
10^2
10^3
10^4
10^5
10^6
Count
PD-L1
Pano + IFN-γ
Pano
IFN-γ

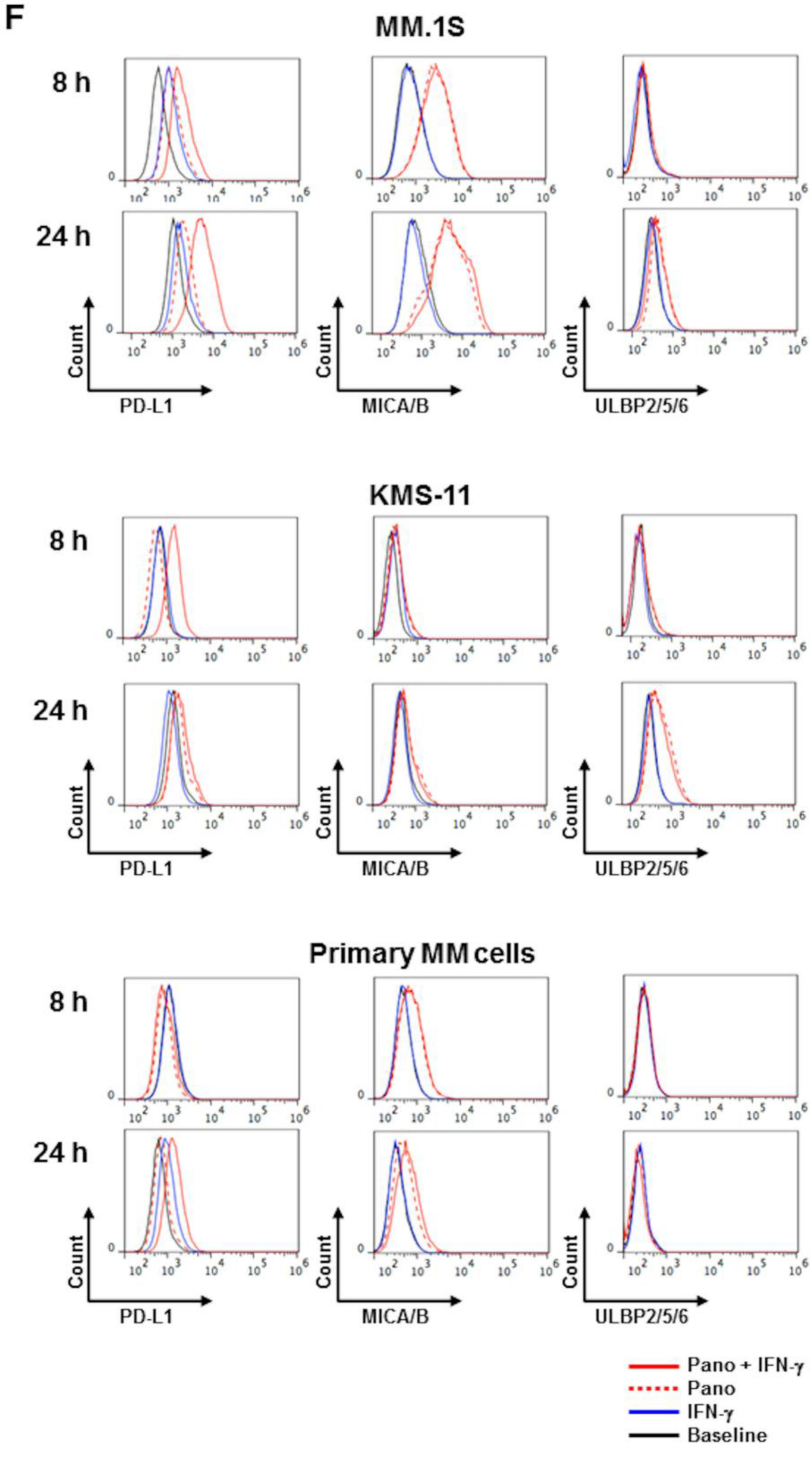

Supplementary Figure 1: (**A**) Surface expression of PD-L1 on MM cells. MM cell lines as indicated were cultured in the presence or absence of IFN-γ for 24 hours. IFN-γ was added at the indicated concentrations. The surface expression of PD-L1 was then analyzed by flow cytometry. MFI of PD-L1 is shown. Base, baseline; IFN, IFN-γ. (**B, C**) Graph presentation of MFI of each condition in Figure 2C (B) and Figure 3A (C). (**D**) Surface expression of PD-L1 on primary MM cells. Primary MM cells from patients #1, 2 and 3 were cultured for 24 hours with or without 25 nM of panobinostat in the presence or absence of 100 U/ml of IFN-γ. The surface expression of PD-L1 was then analyzed by flow cytometry. (**E**) PD-L1 upregulation on MM cells. MM.1S and KMS-11 cells were cultured for 24 or 48 hours with or without 25 nM of panobinostat in the presence or absence of 100 U/ml of IFN-γ. The surface expression of PD-L1 was then analyzed by flow cytometry. (**F**) The surface expression of PD-L1 and Surface expression of PD-L1 and NKG2D ligands on MM cells. MM cell lines and primary MM cells as indicated were cultured with for 8 and 24 hours with or without 25 nM of panobinostat in the presence or absence of 100 U/ml of IFN-γ. The surface expression of PD-L1, MICA/B and ULBP2/5/6 was then analyzed by flow cytometry. Pano, panobinostat.

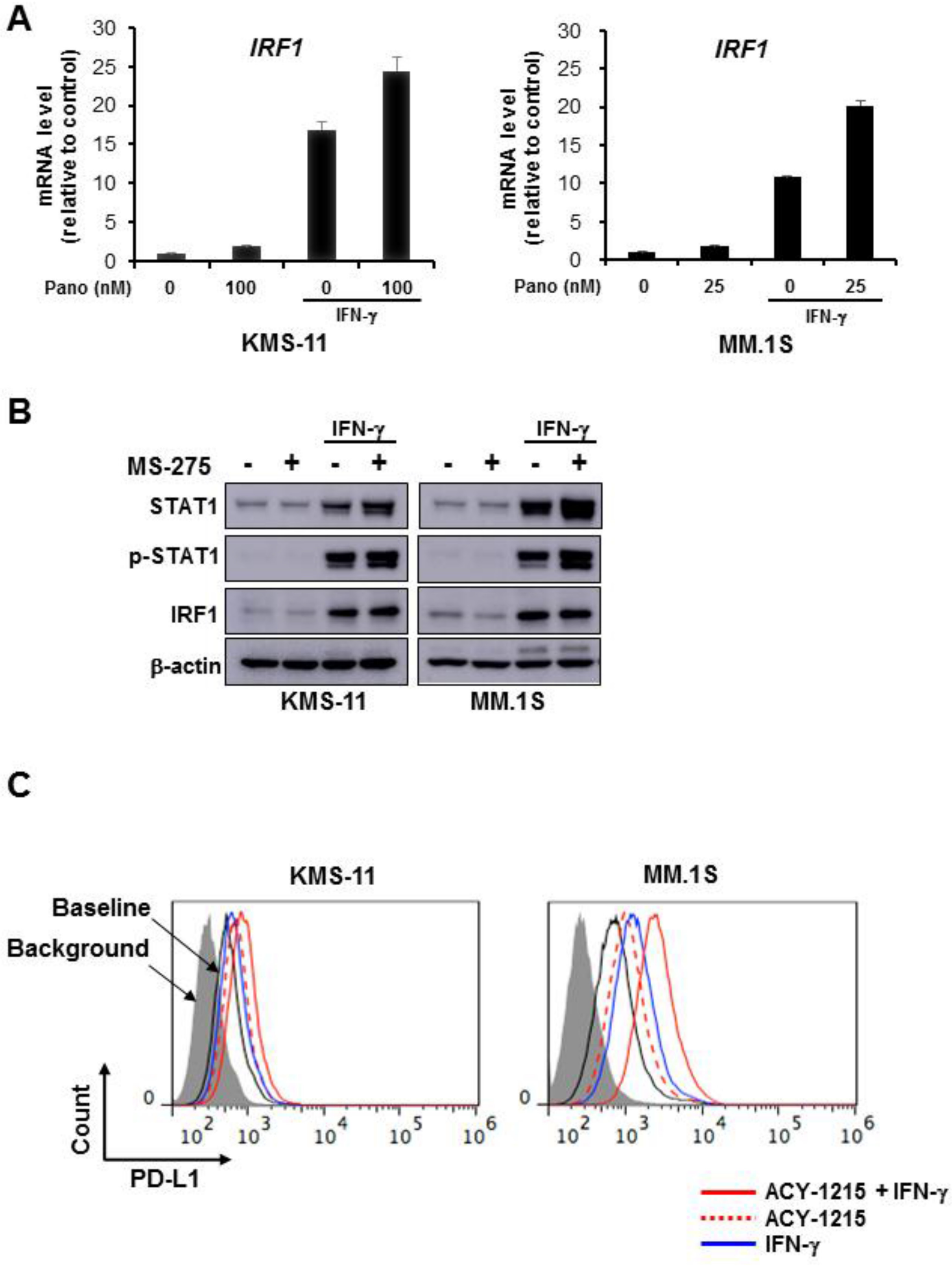

Supplementary Figure 2: (**A**) *IRF1* mRNA expression in MM cells. KMS-11 and MM.1S cells were cultured alone in triplicate for 6 hours or with 100 or 25 nM of panobinostat, respectively, in the presence or absence of 100 U/ml of IFN-γ. *IRF1* mRNA expression was quantified by quantitative RT-PCR. Ratios of *IRF1* over *PPIE* or *GAPDH* mRNA levels were calculated for a normalized target value (defined as 1). *PPIE* or *GAPDH* was used as an internal control. Results were expressed as the mean ± SD. Pano, panobinostat. (**B**) Analysis of the STAT1-IRF1 pathway. KMS-11 and MM.1S cells were incubated for 24 hours with or without MS-275 at 1 μM in the presence or absence of 100 U/ml of IFN-γ as indicated. The cells were then harvested, and STAT1, phosphorylated STAT1 (p-STAT1) and IRF1 protein levels were examined by Western blot analysis. β-actin was blotted as a loading control. (**C**) Surface expression of PD-L1 on MM cells. MM cell lines as indicated were cultured for 24 hours with ACY-1215 at 2 μM in the presence or absence of IFN-γ at 100 U/ml. The surface expression of PD-L1 was then analyzed by flow cytometry.

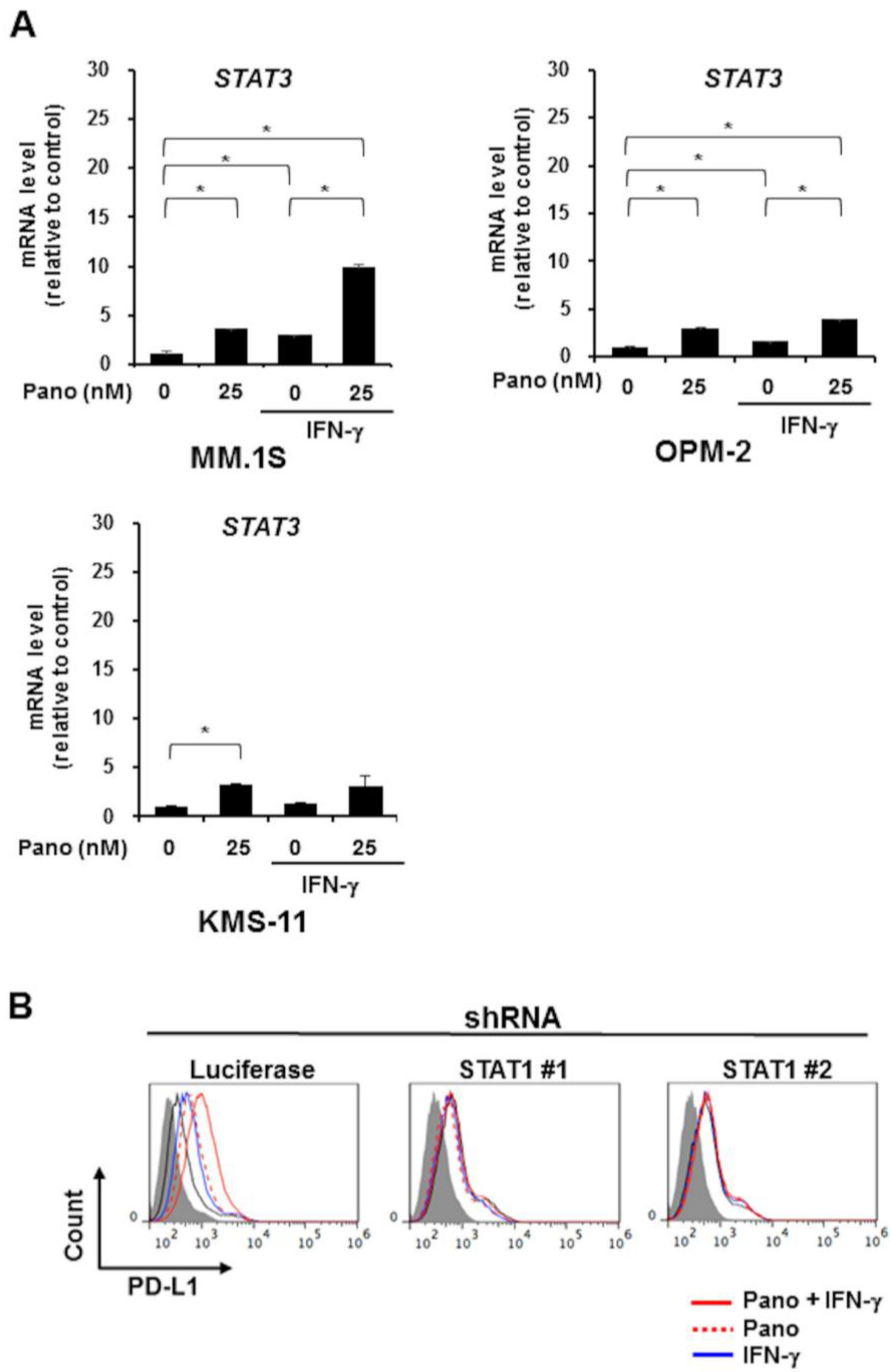

Supplementary Figure 3: (**A**) *STAT3* mRNA expression in MM cells. MM.1S, OPM-2 and KMS-11 cells were cultured alone in triplicate for 24 hours or with 25 nM of panobinostat in the presence or absence of 100 U/ml of IFN-γ. *STAT3* mRNA expression was quantified by quantitative RT-PCR. Ratios of *STAT3* over *PPIE* mRNA levels were calculated for a normalized target value (defined as 1). *PPIE* was used as an internal control. Results were expressed as the mean ± SD. $^{*}p < 0.05$. Pano, panobinostat. (**B**) Effects of *STAT1* gene silencing on PD-L1 expression. *STAT1* shRNA (clones #1 and #2) or control *Luciferase* shRNA were transfected into KMS-11 cells. The cells were cultured for 24 hours with or without 25 nM of panobinostat in the presence or absence of 100 U/ml of IFN-γ. The surface expression of PD-L1 was then analyzed by flow cytometry. Pano, panobinostat.

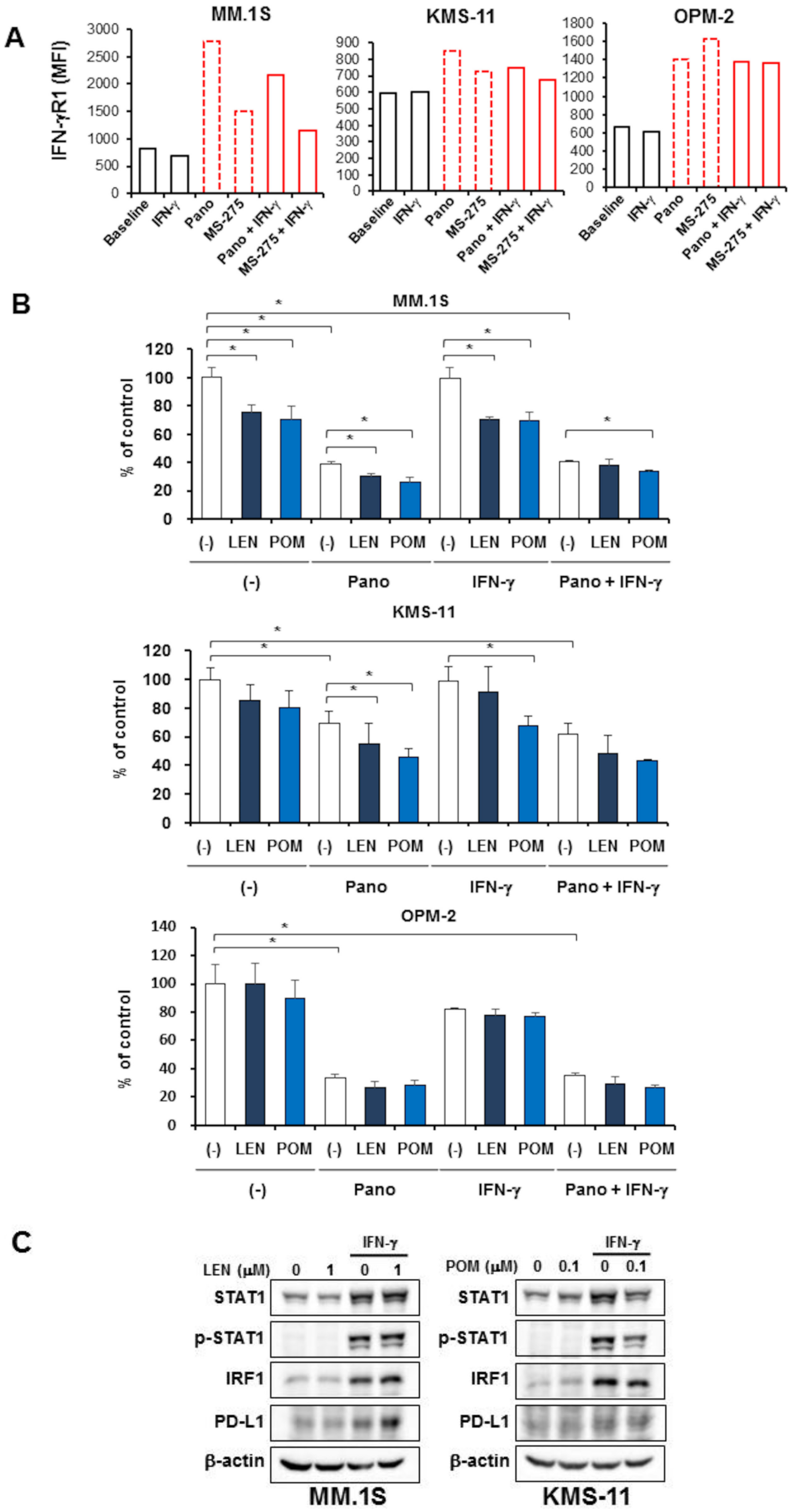

Supplementary Figure 4: (**A**) Graph presentation of MFI of each condition in Figure 5B. (**B**) Viability of MM cells. MM.1S, KMS-11 and OPM-2 cells were cultured with or without 100 U/ml of IFN-γ in the presence or absence of 25 nM of panobinostat, or with or without lenalidomide or pomalidomide in the presence or absence of 25 nM of panobinostat. Lenalidomide or pomalidomide were added at 1.0 or 0.1 μM, respectively. Cell viability was determined by a WST-8 assay after culturing for 2 days. Results are expressed as an optical density (means +/- SD). $^{*}p < 0.05$. (**C**) Analysis of the STAT1-IRF1 pathway. MM.1S and KMS-11cells were incubated for 24 hours in the presence or absence of 100 U/ml of IFN-γ as indicated. Lenalidomide or pomalidomide were added as indicated at 1.0 or 0.1 μM, respectively. The cells were then harvested, and STAT1, phosphorylated STAT1 (p-STAT1), IRF1 and PD-L1 protein levels were examined by Western blot analysis. β-actin was blotted as a loading control.

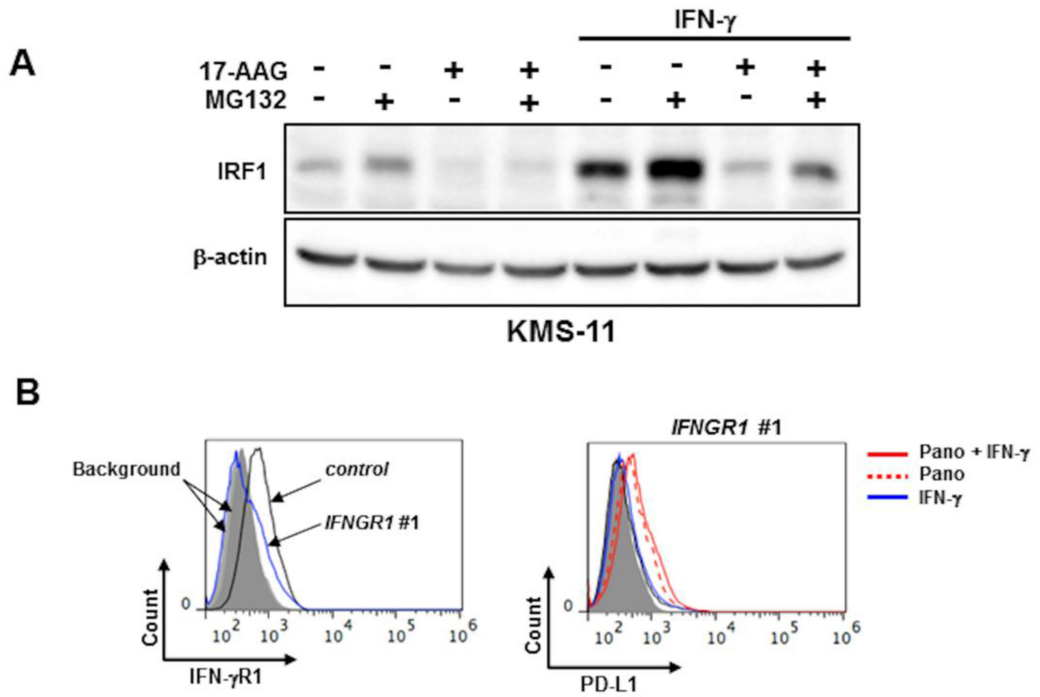

Supplementary Figure 5: (**A**) IRF1 protein levels in MM cells. KMS-11 cells were cultured for 16 hours with or without 17-AAG at 1 μM in the presence or absence of 100 U/ml of IFN-γ as indicated. MG132 was added at 10 μM for the last 4 hours of the incubation period as indicated. IRF1 protein levels were analyzed by Western blot analysis. β-actin was blotted as a loading control. (**B**) *IFNGR1* shRNA (clones #1) or *Luciferase* shRNA (control) was transfected into KMS-11 cells. The knockdown efficacy was examined by flow cytometry. PD-L1 expression on the cells was analyzed by flow cytometry after incubating for 24 hours with panobinostat at 25 nM or IFN-γ at 100 U/ml alone or both in combination.

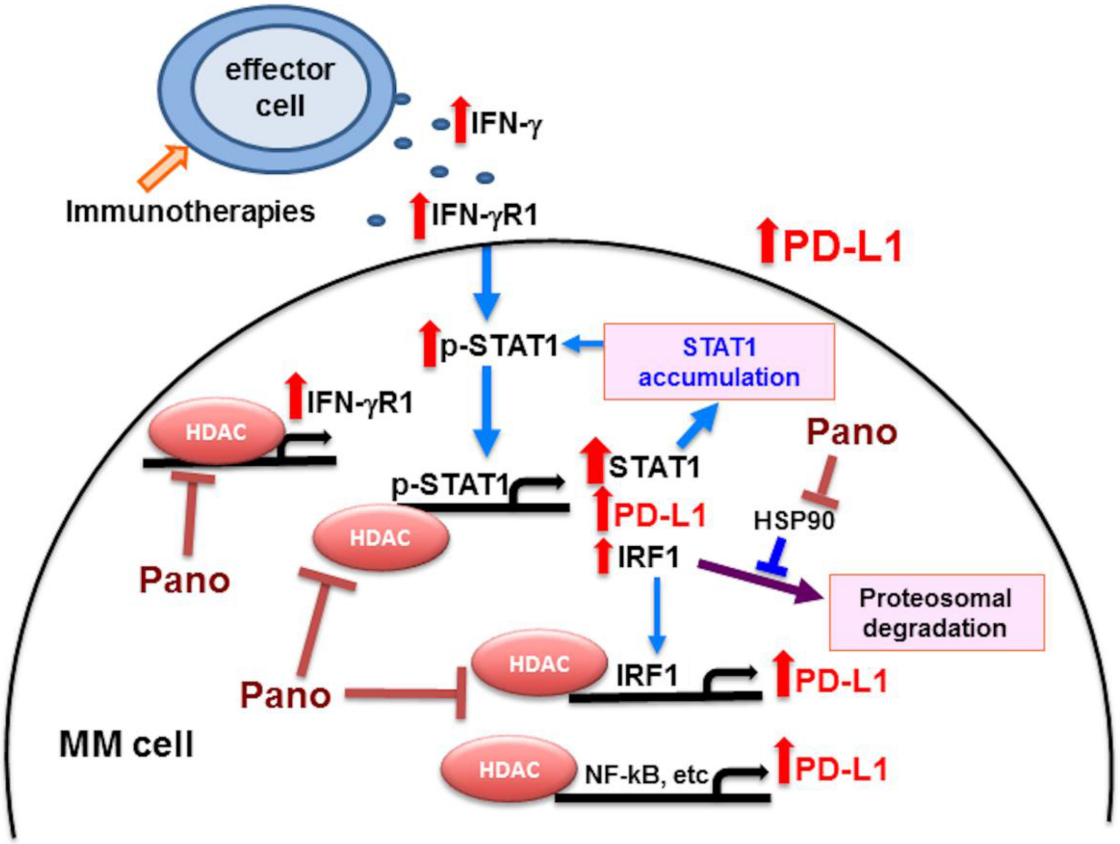

Supplementary Figure 6: In immunotherapies, immune effector cells are stimulated to enhance their IFN-γ production into an ambient tumor microenvironment. IFN-γ plays a vital role in immune responses. Panobinostat enhances IFN-γR1 expression on MM cells, which facilitates the IFN-γR1-STAT1 signaling in a ligand-dependent manner. Phosphorylated STAT1 induces the transcription of its target genes, including *STAT1*, *PD-L1* and *IRF1*, which is further upregulated by HDAC inhibition by panobinostat. Thus, panobinostat substantially increases the total and phosphorylated levels of STAT1 protein while upregulating PD-L1 expression in MM cells in the presence of IFN-γ. However, panobinostat reduces IRF1 protein levels through its proteasomal degradation in the presence of IFN-γ at least in part through inhibition of Hsp90. Besides, panobinostat is able to directly upregulate PD-L1 expression in MM cells through the mechanisms other than IFN-γ–mediated ones. Pano, panobinostat.
